# Cellular Therapy via Spermatogonial Stem Cells for Treating Impaired Spermatogenesis, Non-Obstructive Azoospermia

**DOI:** 10.3390/cells10071779

**Published:** 2021-07-14

**Authors:** Nesma E. Abdelaal, Bereket Molla Tanga, Mai Abdelgawad, Sahar Allam, Mostafa Fathi, Islam M. Saadeldin, Seonggyu Bang, Jongki Cho

**Affiliations:** 1 Institute for Integrative Biology of the Cell (I2BC), CEA, CNRS, Universite Paris-Saclay/Île-de-France, 91198 Gif-sur-Yvette, France; nesma.elsayed-abdelaal-hassan@i2bc.paris-saclay.fr; 2 College of Veterinary Medicine, Chungnam National University, Daejeon 34134, Korea; tanga@o.cnu.ac.kr (B.M.T.); bangsk97@o.cnu.ac.kr (S.B.); 3 Faculty of Veterinary Medicine, Hawassa University, P.O. Box 05 Hawassa, Ethiopia; 4 Biotechnology and Life Sciences Department, Faculty of Postgraduate Studies for Advanced Sciences (PSAS), Beni-Suef University, Beni-Suef 62521, Egypt; maiAli@psas.bsu.edu.eg; 5 Faculty of Medicine, Tanta University, Tanta 31527, Egypt; sahar30839688@med.tanta.edu.eg; 6 Biotechnology Program, Faculty of Agriculture, Ain Shams University, Giza 11566, Egypt; mostafa.fathi61198@gmail.com; 7 Department of Physiology, Faculty of Veterinary Medicine, Zagazig University, Zagazig 44519, Egypt; islamms@zu.edu.eg

**Keywords:** infertility, male, spermatogenesis, spermatogonial stem cells

## Abstract

Male infertility is a major health problem affecting about 8–12% of couples worldwide. Spermatogenesis starts in the early fetus and completes after puberty, passing through different stages. Male infertility can result from primary or congenital, acquired, or idiopathic causes. The absence of sperm in semen, or azoospermia, results from non-obstructive causes (pretesticular and testicular), and post-testicular obstructive causes. Several medications such as antihypertensive drugs, antidepressants, chemotherapy, and radiotherapy could lead to impaired spermatogenesis and lead to a non-obstructive azoospermia. Spermatogonial stem cells (SSCs) are the basis for spermatogenesis and fertility in men. SSCs are characterized by their capacity to maintain the self-renewal process and differentiation into spermatozoa throughout the male reproductive life and transmit genetic information to the next generation. SSCs originate from gonocytes in the postnatal testis, which originate from long-lived primordial germ cells during embryonic development. The treatment of infertility in males has a poor prognosis. However, SSCs are viewed as a promising alternative for the regeneration of the impaired or damaged spermatogenesis. SSC transplantation is a promising technique for male infertility treatment and restoration of spermatogenesis in the case of degenerative diseases such as cancer, radiotherapy, and chemotherapy. The process involves isolation of SSCs and cryopreservation from a testicular biopsy before starting cancer treatment, followed by intra-testicular stem cell transplantation. In general, treatment for male infertility, even with SSC transplantation, still has several obstacles. The efficiency of cryopreservation, exclusion of malignant cells contamination in cancer patients, and socio-cultural attitudes remain major challenges to the wider application of SSCs as alternatives. Furthermore, there are limitations in experience and knowledge regarding cryopreservation of SSCs. However, the level of infrastructure or availability of regulatory approval to process and preserve testicular tissue makes them tangible and accurate therapy options for male infertility caused by non-obstructive azoospermia, though in their infancy, at least to date.

## 1. Male Infertility

Male infertility in humans is defined as the inability to fertilize their female counterpart through unprotected copulation for a period of 12 months and more [1]; the World Health Organization (WHO) has defined infertility as incapability to conceive a child after unprotected, regular sexual intercourse for at least 12 months [2]. At least 30 million men worldwide suffer from infertility, with the highest prevalence found in Africa and Eastern Europe. However, because of the varying opinions with different credibility and older articles, it is quite difficult to provide accurate statistics [3]. Infertility is considered one of the main health problems affecting couples in the reproductive age, affecting about 8–12% of couples worldwide [4], and it was estimated that the prevalence of age-standardized infertility increased by 0.370% in women and by 0.291% in men every year between 1990 and 2017 [5], according to a global burden of disease survey. Infertility is associated with physiological and economic burdens as well as social distress among patients, their families, and healthcare systems; therefore, early detection of the problem and proper management is very important [6,7,8].

Causes of infertility could be attributed to the man only in about 20–30% of couples, [9,10]. Several studies have observed a decline in sperm count over the years, and a meta-analysis conducted in 1992 observed that sperm count has decreased by 50% over 60 years, which was confirmed by another large systematic review that reported that between 1973 and 2011, sperm count has declined by 50–60% [11,12,13,14]. Male infertility is attributed to the diminished quantity or quality of the ejaculate, and when sperm are not found in the ejaculate, the case called azoospermia. Azoospermia can be caused by obstructive reasons such as congenital bilateral absence of vas deferens, congenital obstruction of the duct system, incomplete patency of the vas deferens, or slow maturation of spermatozoa in the epididymis [15,16]. Non-obstructive azoospermia can be caused by congenital causes such as Kallmann syndrome, Klinefelter syndrome, genetic endocrinopathy, cryptorchidism, microdeletions of the Y chromosome, anorchia, and idiopathic mild androgen insensitivity syndrome [17]. Acquired causes of non-obstructive azoospermia include varicocele, which is the most common cause of infertility in men [18], germ cell tumors, acquired hypogonadotropic hypogonadism (HGH), exogenous factors such as exposure to chemotherapy or radiation or cytotoxic drugs, recurrent urogenital infections, and other systemic diseases and surgeries that can affect spermatogenesis or male endocrine functions [19]. Idiopathic factors include unknown causes of male infertility and account for approximately 30–50% of infertile male cases [20], including environmental factors such as exposure to toxic chemicals [21], lifestyle factors such as obesity [22], smoking [23], and alcohol consumption [24], and psychological factors such as emotional stress, anxiety, and depression [25,26]. In this review, we shed the light on the potential of treating infertility caused by non-obstructive azoospermia through cellular therapy using spermatogonial stem cell transplantation.

### 1.1. Azoospermia

Azoospermia is defined as the absence of spermatozoa or sperm in the semen; it is considered the cause of infertility in approximately 10% to 15% of fertility in men [27]. This could be due to pretesticular, testicular, or post-testicular causes. Pretesticular causes of azoospermia include hormonal problems of the hypothalamic-pituitary-gonadal axis [28]. Testicular causes include abnormalities in the spermatogenesis process occurring inside the testis, and post-testicular causes include obstructions of ducts in the male reproductive system [29]. Pretesticular and post-testicular causes are usually treated, and fertility can be restored, while testicular causes are usually not reversible, with low success rates of several interventions. Pretesticular causes and testicular causes are considered to be non-obstructive causes of azoospermia, while post-testicular causes are considered to be the obstructive causes of azoospermia.

#### 1.1.1. Pretesticular Causes

Pretesticular causes are usually due to pathological endocrinal problems in origin, and they are also called secondary testicular failure, affecting up to 3% of infertile males [30]. HGH is a rare cause of infertility in men and is characterized by a decline in testosterone, luteinizing hormone (LH), and follicle-stimulating hormone (FSH), which can be congenital or acquired, and it is considered as one of the treatable causes of male infertility. Causes of HGH include pituitary gland tumors, pituitary gland traumas, Kallmann syndrome, and anabolic steroid use [31].

Kallmann syndrome, the most common cause of congenital HGH, is caused by abnormalities in the midline cranial structures, and is characterized by decreased secretion of gonadotropin-releasing hormone (GnRH) from the hypothalamus resulting from failed migration of GnRH-releasing neurons during development to the olfactory lobe [32]. The disease is X-linked, and it has been reported that it is caused by mutations in the KAL gene represented on Xp22.3 position [33]. Androgen resistance is another congenital cause of HGH, which occurs due to several mutations of the androgen receptor gene on the X chromosome [34,35], affecting males and females leading to infertility; although androgen resistance is complete in females, it is partial in males and both results in infertility. Testosterone levels can increase, decrease, or remain normal according to the severity of genetic defects, and impaired androgen receptor is the primary cause of decreased or absent sperm in 40% of infertile men [36]. Acquired causes of HGH include tumors of the pituitary gland, panhypopituitarism, trauma of the pituitary gland, and using anabolic steroids [37]. Exogenous testosterone inhibits the secretion of gonadotropins by a negative feedback loop, leading to the inhibition of the hypothalamic-pituitary-gonadal axis [38]. Using anabolic steroids excessively increases androgen levels, resulting in decreased levels of intratesticular testosterone levels and decreased FSH levels, resulting in impaired spermatogenesis, which leads to azoospermia and infertility in men [39]. Hyperprolactinemia causes HGH by inhibition of GnRH secretion in the hypothalamus caused by excess prolactin, which also causes a decline in FSH and LH hormone levels [40,41]. Hyperprolactinemia could be a consequence of using certain medications such as antihypertensive drugs, tricyclic antidepressants [42], or due to certain tumors such as pituitary gland tumors, including prolactin-secreting microadenoma, and prolactin-secreting macroadenoma, which leads to the destruction of the anterior pituitary gland and increased prolactin secretion, which could also be idiopathic [43].

#### 1.1.2. Testicular Causes

They are identified as a primary testicular failure caused by internal abnormalities affecting the process of spermatogenesis inside the testis, which could result from developmental errors, genetic mutations, and the genotoxic effect of certain medications. Varicocele is a medical condition resulting from dilatation of veins draining the testis and belonging to the pampiniform plexus, which affects approximately 25% of males with decreased sperm count and about 15% of normal males [7]. It is hypothesized that the mechanism by which varicocele affects the testis is to increase the temperature of the testis and production of reactive oxygen species resulting from the stasis of venous blood in the pampiniform plexus [44].

Cryptorchidism, also known as undescended testes, is one of the most common causes of azoospermia in men; it affects approximately 25% of patients with unilateral cryptorchidism, and 80% of patients with bilateral cryptorchidism [45]. Early treatment is important to decrease infertility risk, and success rates of treatment depend on the initial position of the testis [46].

Testicular torsion is defined as testicular spinning inside the scrotum, twisting blood vessels supplying it, leading to decreased blood supply and resulting in pain and swelling. Testicular torsion is considered a pediatric emergency that sometimes requires surgical intervention to avoid loss of testicles that would lead to decreased spermatogenesis and infertility [47,48]. It affects approximately 45 of every 1000 males under 25 years of age per year [49].

Orchitis is the inflammation of the testis, which is characterized by an accumulation of exudate inside and outside seminiferous tubules leading to its damage, which could be due to systemic or local inflammation, autoimmune and idiopathic origin, or infection by certain types of bacteria such as *Chlamydia trachomatis*, or mumps virus [50]. Orchitis is considered the most common manifestation of mumps infection in men; it can affect the testis unilaterally in 67% cases, and it could be bilateral in 33% of affected men, in 36% of them, resulting in testicular atrophy, which would lead to infertility in approximately 13% of patients [50,51].

Several genetic abnormalities can cause infertility in men, including Klinefelter syndrome, 47(XYY) syndrome, XX male syndrome, and Y-chromosome microdeletions [52].

Importantly, cytotoxic drugs used in tumor therapy can lead to non-obstructive azoospermia. Although chemotherapy or radiotherapy have shown great advances in recent years with increased five-year cancer survival rates among children and adults, it could result in infertility as a long-term side effect, and 24% of treated cancer patients could suffer from impaired spermatogenesis and infertility in the long term [53].

Previous data showed that the testis is radiosensitive [54], and the degree of gonadal damage depends on the radiation delivery method and dosage. Impaired spermatogenesis and loss of germ cells can result from direct treatment of the testis or adjacent tissue by radiation [55]. It was observed that treatment of cancer with fractionated radiation for 3 to 4 weeks would result in delayed recovery of gonadal damage and lead to infertility [56]. High doses of radiation could also result in permeant azoospermia because it can kill all spermatogonial stem cells (SSC) [57,58], while low doses were associated with lower rates of gonadal damage, and spermatogenesis recovery [59,60].

Chemotherapeutic agents, especially alkylating medications, could also lead to a decrease in sperm count and infertility. Chemotherapy could result in 10- to 100-fold reduced sperm count, damage of Leydig cells, and azoospermia [61,62]; chemotherapy without or in addition to radiation is responsible for infertility in 60% of men treated at sites below their diaphragm [63]. The permanence and recovery rate of infertility associated with chemotherapy depends on the duration of treatment, addictive drugs, and doses of medications used. To date, it is difficult to accurately predict the risk factors of azoospermia, whether it is temporary or permanent, and recovery rates of spermatogenesis [64].

#### 1.1.3. Post-Testicular Causes

Post-testicular causes are considered obstructive azoospermia, which usually results from obstructions of ducts carrying sperm or impaired ejaculation. The absence of vasa deferentia is a congenital disease characterized by bilateral absent vas deferens, accounting for 6% of obstructive azoospermia patients and about 1% of infertile patients [65]. The absence of vasa deferens can result from abnormal cystic fibrosis transmembrane regulator gene (CFTR) [66], and it has been reported that CFTR mutations are present in 80% of men with bilateral absence of vas deferens, and 43% of those with unilateral vas deferens. The absence of vas deferens can also result from the abnormal differentiation of the mesonephric duct [67,68]. Obstruction of vas deferens is another cause of obstructive azoospermia. The main cause of obstructed vas deferens is a vasectomy procedure that is usually carried out for elective sterilization [69]. Another cause of obstruction is an accidental injury during inguinal hernia repair [70], or it could result from an inflammatory reaction against polypropylene mesh used during inguinal hernia repair [71].

Ejaculatory duct obstruction can also lead to obstructive azoospermia and infertility in about 1 to 5% of cases [72], which could be congenital due to Mullerian or Wolffian cysts, or acquired due to trauma, seminal vesicle calculi, or calcification of the prostate, which could affect one or both ejaculatory ducts [73,74]. Ejaculatory dysfunction includes a group of disorders with impaired ejaculation, such as premature ejaculation, retrograde ejaculation, anejaculation, and delayed ejaculation. The prevalence of ejaculatory dysfunction in infertile men is below 2%, and it is not considered a common cause of infertility in men [75,76]. Of note, infertility caused by obstructive azoopermia can be treated through some surgical approaches and using assisted reproductive techniques such as in vitro fertilization (IVF), intracytoplasmic sperm injection (ICSI), or even testicular sperm aspiration (TESA) and testicular sperm extraction (TESE) [30,77].

#### 1.1.4. Other Causes of Male Infertility

In addition to cytotoxic drugs, some drugs could affect men fertility, leading to reduced sperm count. Sirolimus, an immunosuppressive drug, can lead to decreased sperm count and motility as a result of seminiferous tubule dystrophy and decreased intratesticular testosterone levels. Antihypertensive drugs such as calcium channel blockers and beta-blockers affect erectile function and testosterone levels. Serotonin reuptake inhibitors, which are antidepressant and antipsychotic drugs, could lead to hyperprolactinemia, which results in azoospermia and infertility, while diuretics such as aldosterone can lead to infertility by inhibiting testosterone biosynthesis, inhibiting androgen binding to target cells, and impairing sperm motility [78].

## 2. Spermatogonial Stem Cell as New Option to Treat Impaired Spermatogenesis

**Figure 1:**
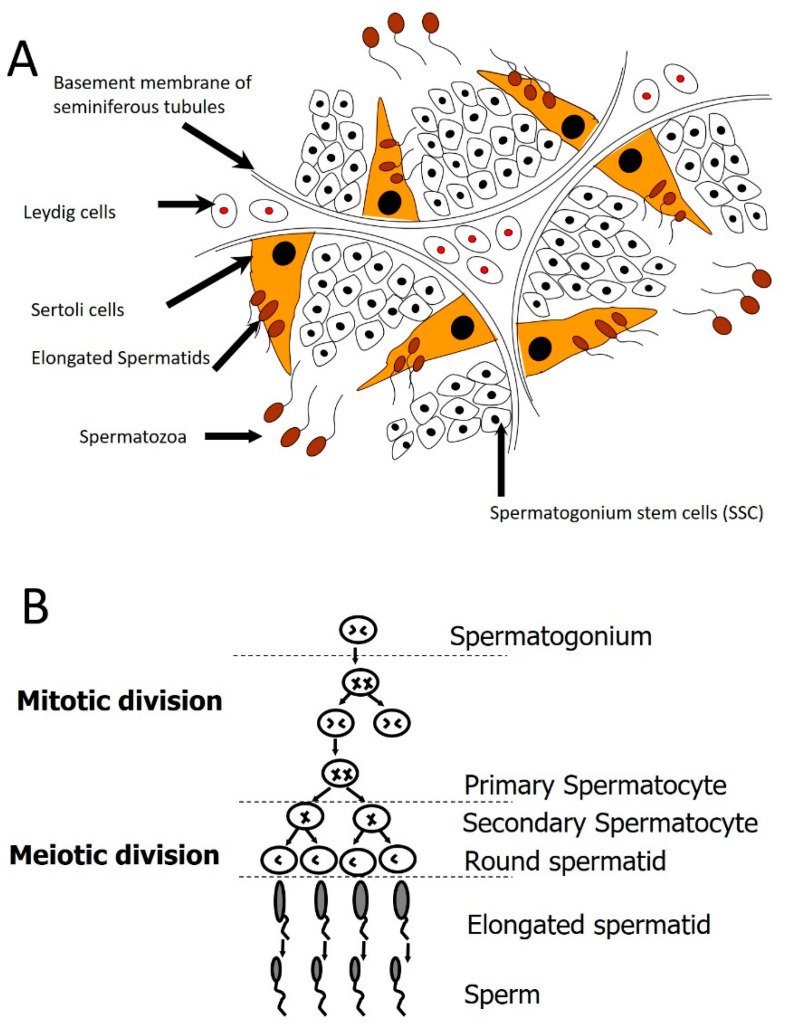
The process of spermatogenesis. (**A**) A cross-section in the testis, showing the process of spermatogenesis occurring in the seminiferous tubules with the aid of Sertoli cells. (**B**) After puberty, spermatogonial stem cells proceed into infinite mitotic and meiotic divisions resulting in millions of spermatozoa [97].

Spermatogenesis starts after puberty, in which spermatogonia undergo mitotic and meiotic divisions, resulting in haploid spermatids and spermatozoa (Figure 1). The final steps for spermatozoa maturation occur in the epididymis. Since spermatogenesis is considered a stem cell-based mechanism [79], one way to treat male infertility caused by impaired spermatogenesis is through stem cell transplantation (Figure 1). This is because stem cells are unspecialized cells that can self-renew, regenerate, and differentiate into other cell types [80]. SSCs can restore spermatogenesis when spermatogonial cells get damaged and/or depleted [79,81]. Therefore, stem cell transplantation represents a promising technique for reviving spermatogenesis in cancer patients and other patients who suffer from impaired spermatogenesis [82,83]. Patients with cancer who undergo radiation or chemotherapy suffer from male infertility as a consequence of the treatment side effects. Therefore, fertility maintenance has become a major concern in the care of prepubertal boys receiving cancer treatment. Accordingly, testicular biopsy is performed, and autologous SSCs are isolated and cryopreserved before cancer treatment, followed by stem cell transplantation intra-testicularly [82,84]. On the other hand, other stem cell types have also been used for azoospermia treatment. For instance, mesenchymal stem cells (MSCs), embryonic stem cells (ESCs), very small embryonic-like stem cells (VSELs), and induced pluripotent stem cells (iPSCs) are obtained from normal somatic cells [3,83].

SSCs are characterized by their capacity to maintain the self-renewal process and possess the pluripotency [85,86,87] where they can differentiate as any stem cell type; the basis of spermatogenesis and male fertility are SSCs, which are rare and constitute only 0.03% of all germ cells in rodent testicles, while SSCs are greatly outnumbered by the differentiating spermatogonia, spermatocytes, spermatids, and sperm produced by them [88]. This equilibrium retains the population of stem cells and satisfies the proliferative requirement of the testis to generate millions of sperm daily [89]. The development of this unique cell form provides novel biotechnological solutions to existing human issues as a result of ongoing and comprehensive SSC biomedical research.

SSCs originate from gonocytes in the postnatal testis that originate from long-lived primordial germ cells (PGCs) during early embryonic development. PGCs are a transient cell population that are first detected in the epiblast stage embryo as a small cluster of alkaline phosphatase-positive cells at around 7–7.25-days post coitum (dpc) in rodents, while in humans, they were noticed in the yolk sac wall, close to the allantois around 24 dpc in humans [90] and depends on the extraembryonic ectodermal expression of BMP4 and BMP8b [86,87]. The PGCs are passively swept out of the embryo during the development of the allantois until they begin to migrate through the hindgut to enter the oblivious gonadal ridge in mice between 8.5 and 12.5 dpc, and between 29–33 dpc in humans [91]. During the migration pattern process, approximately 3000 PGCs colonize the genital ridges [92]. PGCs give rise to gonocytes that are enclosed in testicular cords constituted by Sertoli precursor cells and peritubular myoid cells in the male gonads at approximately 13.5 dpc. The general definition of gonocytes can indeed be subclassified into mitotic (M)-prespermatogonia, T1-prospermatogonia, and T2-prospermatogonia [93]. M-prespermatogonia is found in the middle of the testicular cords, farther from the basement membrane, and when they become T1-prospermatogonia and reach G0 mitotic arrest, they tend to proliferate until around 16.5 dpc of rodent development [94]. Gonocytes begin proliferating and migrate to the seminiferous tubules of the basement membrane within the first week after birth (marking their transition to T2-prospermatogonia) [95]. The first round of spermatogenesis occurs in T2-prospermatogonia that colonize the basement membrane and the initial pool of SSCs that sustain spermatogenesis during post-pubertal life [93,96].

It is crucial to understand SSCs in the sense of the spermatogenic lineage they generate in an attempt to comprehend the regulation of SSCs. Spermatogonia, found on the basement membrane of seminiferous tubules, are diploid germ cells. Spermatogenic lineage development differs between rodents and humans [98]. In rodents, three types of spermatogonia were originally identified based on their nuclear morphology [99]. Type A spermatogonia are known to be the most primitive because the nucleus lacks heterochromatin, a general feature of undifferentiated cells. There is a reasonable quantity of heterochromatin in the nuclei of intermediate-type spermatogonia and a significant amount of heterochromatin in type B spermatogonia, suggesting a more differentiated state [100]. Studies have shown that undifferentiated type A spermatogonia can indeed be subcategorized into spermatogonia of A single (As), A paired (Apr), and Aligned (Aal), which vary only in their topographical configuration on the basement membrane of the seminiferous tubule [101,102]. It generates an Apr when an As spermatogonium divides, that either (i) perform cytokinesis to produce two new As spermatogonia or (ii) persists linked by an intercellular cytoplasmic bridge and generates a chain at the next division of four Aal spermatogonia. Additional cell divisions contribute to the development of 8, 16, and occasionally 32 Aal spermatogonia chains; chains of 4–16 Aal are usually deemed dedicated to the mechanism of differentiation. Therefore, the pool of stem cells contains As and some Apr spermatogonia. Some may have argued that stem cells could expand to larger clones [103,104]. The smaller chains of As, Apr, and four Aal spermatogonia were uniformly distributed around the seminiferous epithelium. Larger Aal chains (8, 16, and 32) differentiate A1 spermatogonia between stages IV and VIII of the seminiferous epithelium, and at stage IX, these eventually lead to A2 spermatogonia. Therefore, unlike undifferentiated spermatogonia, differentiating spermatogonia A1, A2, A3, A4, intermediate, and B divide synchronously and occur at particular stages of the seminiferous epithelium. B spermatogonia generate primary spermatocytes that advance into meiosis, and two meiotic divisions contribute to the development of secondary spermatocytes and haploid spermatids, which induce morphological changes in 16 stages and gradually become sperm close to completion from the seminiferous epithelium [105,106].

In humans, the A0/A1 model is an alternative to the As model of SSC self-renewal, which is very close to the model of Adark and Apale used in non-human primates to characterize stem cell potency [98]. Adark spermatogonia act as reserve stem cells, while Apale spermatogonia are the self-renewing stem cells. There is only one generation of type B spermatogonia before differentiation into spermatocytes, which makes human spermatogenesis less efficient than in rodents [107]. A0 spermatogonia were identified as single cells or pairs of cell types found in the seminiferous epithelium during all phases. In these cells, mitotic measurements were seldom found, and therefore they were assumed to not lead to stable spermatogenesis as reserve stem cells. These reserve stem cells are triggered only by harmful threats, such as radiation, and disable spermatogenesis. A1–A4 spermatogonia are part of the functional stem cell pool. They inevitably lead either to new A1 spermatogonia (described as self-renewal) or intermediate spermatogonia if A4 spermatogonia split (described as differentiation) [108,109,110].

SSCs locate within a standardized microenvironment called ′niche′, which, by maintaining SSC self-renewal, quiescence, pluripotency, and differentiation, controls testicular homeostasis [111]. In the atmosphere of stem cells that govern cell fate, a stem cell niche is made up of cells, extracellular fluids, and local soluble factors. The basal region of the seminiferous tubules containing Sertoli cells and peritubular myoid cells is the structural basis for the SSC niche in mammalian testes [112]. Sertoli cells produce growth factors that stimulate self-renewal (Glial cell line-derived neurotrophic factor, and basic fibroblast growth factor) and differentiation (activin A, bone morphogenetic protein 4, and stem cell factor) of the SSCs, as well as other factors to maintain the microenvironment such as chemokine (C-X-C motif) ligand 12, vascular endothelial growth factor A, inhibin βA, NOTCH and WNT5A [113,114]. Sertoli and peritubular myoid cells simultaneously release the constituents of the basement membrane to which SSCs are linked through binding proteins [115]. Sertoli cells sustain SSCs and differentiate germ cells in addition to supporting spermatogenesis by supplying nutrients and modulating external signals [116]. Transplantation of normal Sertoli cells into the testes of infertile mutant patients with Sertoli cell deficiency and successful induction of spermatogenesis by recipient-derived spermatogonia have shown the significance of Sertoli cells in germ cell proliferation [117,118]. A protective blood-testis barrier (BTB) is a close junction between adjacent Sertoli cells, which divides the seminiferous epithelium into basal and adluminal compartments and plays a vital role in controlling germ cell proliferation [119]. BTB ensures preferential circulation of substances between the luminal fluid, bloodstream, and interstitial fluid, providing an immune-privileged environment in the adluminal compartment of the seminiferous tubules for haploid germ cells [119].

### 2.1. Spermatogonial Stem Cell Transplantation for Regeneration

Brinster and colleagues were the first to identify a technique for transplanting SSCs [120]. Fertility preservation in rodents after SSC transplantation indicates that this technique could have medical application in humans [83,121]. The critical points are the success of SSCs storage (good cryopreservation) and transplantation, and when transplanted, induction of spermatogenesis [122]. Testicular biopsy and cryopreservation are promising tools for people with cancer and undergoing cancer treatment. Scientists were able to propagate SSCs in humans in vitro. However, more research is required to overcome some challenges and improve outcomes [123]. Lim et al. proved that SSCs found in the testes of patients with non-obstructive azoospermia can be extracted, isolated, and propagated in vitro via a highly efficient culture system, resulting in the differentiation of germ cells with developmental potential [124].

#### 2.1.1. Testicular Biopsy/Tissue

Under sterile conditions, a biopsy is obtained from the testis of children with cancer before undergoing treatment or from adult patients with azoospermia, regardless of the cause. An alternative method for biopsy is to obtain testicular tissue in humans. In contrast, in the case of animals such as rodents, whole testes are obtained and preserved in sterile Hanks′ balanced salt solution (HBSS) [125]. To obtain a single-cell suspension from the young, the testicular tissue must be digested via enzymatic digestion by mixing it with trypsin–EDTA solution and DNase I solution. The cell suspension is washed with HBSS after passing through a 40-m pore cell strainer, and then centrifuged (cool centrifuge) to obtain a pellet. In the case of adult animals, the testes are mixed with collagenase solution and DNase I solution, mixed until the separation of the seminiferous tubules, added in HBSS in ice until complete sedimentation occurs, the supernatant discarded, the tubules washed with HBSS to remove the interstitial cells, and then complete the same steps performed in case of young cases until the pellets are obtained via cool centrifugation [126,127]. A similar technique has been used in fish, such as *Brycon orbignyanus* [128].

#### 2.1.2. SSCs Isolation

Goodyear and Brinster established a complete protocol for SSCs in mice, including isolation, culturing, expansion, and eventually transplantation [126,127], while Jon Oatley designed a protocol for cryopreservation and thawing [129]. The process of spermatogonial stem cell isolation starts from the digestion (type I collagenase and trypsin) of the obtained testicular tissue or the biopsy to obtain a single cell suspension via mechanical disruptions or enzymes such as trypsin, as discussed above. This can be done via Percoll isolation, which is a highly efficient tool used for separation depending on density gradient centrifugation [125,130]. Therefore, it is used to obtain SSCs via a few simple steps. After obtaining the pellet, HBBS is added for pellet resuspension, and diluted until it reaches 20 × 10^6^ per volume of 5 mL. The cell suspension layer is obtained above Percoll solution. The cell pellets are mixed with PBS-S, and the cell concentration is calculated. The cell content is diluted to 5 × 10^6^ cells/mL. Then the process of cell sorting is performed via magnetic-activated Cell Sorting (MACS) to select Thymus cell antigen 1 (Thy1+) or octamer-binding transcription factor 4 (Oct4+) or the promyelocytic leukaemia zinc finger (PLZF+) cells which are specific for SSCs [125,130,131]. Thy1 antibody-conjugated microbeads are added to the cell suspension (cell pellet with PBS) containing the SSCs with specific dilutions, and then incubated at a specific temperature for a certain time. After sterilization and preparation of the MACS column, the cell suspension containing the Thy1+ antibody microbeads are allowed to pass, and the solution containing the Thy1+ cells in serum-free medium is eluted, and the cell concentration calculated. Now we have Thy1+ cells, and by this, the process of SSC isolation is completed [115,117]. Moreover, a discontinuous density gradient is also used in the process of SSC isolation in fish, such as *Brycon orbignyanus* [128].

#### 2.1.3. SSCs Culturing and Expansion

After isolation of SSCs or after preservation, culture and expansion are required to propagate the cells. Preparation of a primary culture of SSCs is done via preparation of mitomycin-treated STO feeders [132], washing them with HBBS, and adding the SSCs either freshly isolated or cryopreserved. Then, 0.5–1 × 10^5^ Thy1+ cells are added in each plate in 12-well tissue culture plates, and SFM, GDNF, and bFGF are added. The tissue culture plate is placed in a CO_2_ incubator at 37 °C, and the medium is changed every 2–3 days. Then, the process of expansion is performed for the obtained undifferentiated SSC_S_ by washing with PBS and then using trypsin–EDTA for dissociation; the cells are incubated in a CO_2_ incubator, the effect of trypsin is stopped by adding FBS, and cell suspension is obtained by mixing and pipetting, followed by centrifugation to obtain the pellet, SFM, centrifugation, and resuspended again in SFM, GDNF and bFGF. Finally, the suspension is added to the STO feeder layer [127,129]. In humans, the xeno-free and feeder-free culture systems are required for clinical applications [133,134]. For instance, matrigel-based and hydrogel-based culture systems were successfully reported for mice SCC culture [135,136]. While in human, three-dimensional culture system using agarose gel was used to improve the differentiation of SCC to spermatocytes and spermatids [131]. Hydrogel-coated or gelatin-coated dishes together with growth factor (GDNG, bFGF, GFRA1-Fc fusion protein, NUDT6, TGFB, EGF, and LIF) were used for propagation of human GPR125^+^ spermatogonia [137,138,139,140].

#### 2.1.4. SSCs Transplantation

SSC transplantation requires the preparation of recipients. The previous protocols were about the preparation of SSCs from donors. Whether they are autologous or allogeneic or xenogeneic depends on the study purpose and the aim of protocol execution. The initial trials showed the survival of human SCC in mice testis after six months of injection. However, there was no human sperm resulted from this trial [141]. Furthermore, SCC was successfully subsided to the seminiferous tubules after injection into azoopsermic mice [142]. Successful completion of SSC transplantation and retrieval of sperm was reported after 12 months of SCC injection in irradiated dog testis [143]. Recently in the monkey, SSC transplantation partially restored the functional sperm production when testis was irradiated before and after puberty [144]. An important step towards application in humans. In mice, preparation of the recipients is performed via injection of busulfan intraperitoneally to remove the endogenous germ cells in the recipient mice, the injection occurs in bodyweight and species-dependent doses. Animals are left for six weeks to guarantee complete depletion of spermatogenesis before the process of SSC transplantation, to be able to assess and judge that the produced spermatogenesis comes from the transplanted SSCs [129].

**Figure 2:**
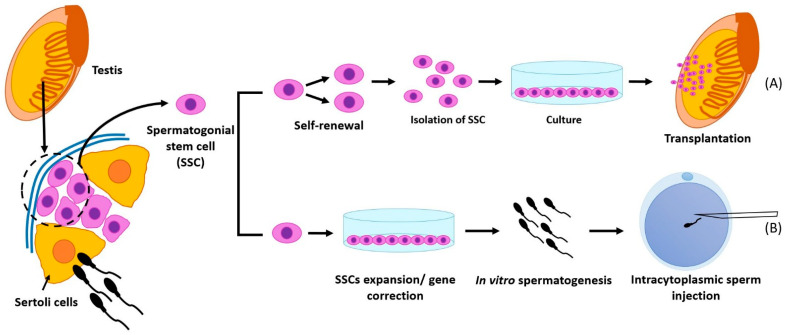
Schematic illustration of spermatogonial stem cells (SSCs) as treatment option for male infertility, (**A**) Transplantation of SSCs into infertile testis after in vitro propagation, (**B**) Application of spermatozoa in intracytoplasmic sperm injection after in vitro spermatogenesis of SSCs.

For SSC transplantation, there are two cases; the first case is wherein the whole testis or the testicular tissue is freshly obtained, and therefore the preparation protocol is needed, while the second case is wherein the cells are already isolated and cultured. In the case of tissues of the whole testis, the tunica albuginea should be removed, the testes added in HBBS, collagenase for separation of the seminiferous tubules, HBBS, trypsin-EDTA added for digestion and cell dispersion, and centrifugation, washing, and resuspension in SFM. In the second case of the culture, a cell suspension is directly performed in SFM. Then, both cases complete the same pathway with the addition of trypan blue for easy gross monitoring during injection, then directly microinjecting the cells in the seminiferous tubule lumen, followed by monitoring and keeping the transplanted recipient males for an additional eight weeks to allow for robust donor-derived spermatogenesis [129]. Figure 2 illustrates the sequences for SCCs transplantation. In contrast, in fish, a simple protocol was designed to perform SSC transplantation from one species to another, that is, from *Brycon orbignyanus* (donor) to *Astyanax altiparanae* (recipient). This was done via enzymatic digestion of the testes, then isolation by density gradient, adding busulfan for germ cell suppression, labeling the SSCs with PKH26, and injecting the recipient fish with SSCs via the urogenital papilla. Twenty-one days after transplantation, sperm were detected in the lumen [128]. The various types of culture media used and the level of achievement in testicular tissue culture in different species are summarized in Table 1.

### 2.2. In Vitro Spermatogenesis Using Spermatogonial Stem Cells

Testicular spermatogonial tissues from a prostate cancer patient were cultured for 27 days and developed into spermatids [92]. On the other hand, the culturing of germline spermatids cells, from testicular tissue biopsies in obstructive azoospermia patients, provides new hope that immature spermatids could mature into mature sperm via in vitro spermatogenesis (IVS) [156] (Figure 2). Similar findings have also been reported by Cremades et al. (1999), where spermatids from non-obstructive azoospermia have been successfully cultured, resulting in IVS, and round spermatids have developed into elongated spermatids [93]. Recently, the Vero cell culture system has been complemented to enhance IVS by supplementing culture media with 50% boar rete testicular fluid or in human synthetic oviduct fluid and 10% human serum, improving IVS rate to 10%, and yielding haploid sperm [94]. And, recently, through the development of an organotypic culture system, IVS achieved from pre-pubertal human testicular tissue (immature spermatids) has been reported from 16 days of culture, resulting in round spermatids [95].

The milestone progress in in vitro spermatogenesis using SSCs from cryopreserved testicular organs was in 2011 using serum-free gas-liquid interface culture in mice [153]. Efforts were made in different species, humans, and Bama minipigs, and various levels of success were attained (Table 1). For the IVS to achieve the required fertility, in addition to spermatid elongation, additional evidence of fertility indicators must be obtained in terms of genetic and epigenetic fertility in haploid cells. Perhaps, it is to be demonstrated and clinically proven [10]. On the other hand, the scarcity of spermatid tissue to perform IVS for the long term and the limitation of available data will not allow conclusive remarks regarding its success. Some trials have been conducted to protect spermatids against membrane damage and to augment the motility of differentiated spermatozoa. Dumont et al. used retinol (vitamin A) to culture mouse prepubertal testicular tissue and found an improvement in the differentiation of SSCs into motile spermatozoa after culturing for 30 days [157]. Interestingly, IVS was improved in rats by supplementing the culture medium with hormones (testosterone, thyroxin, FSH, and LH), antioxidants (glutathione and ascorbic acid), and lysophospholipids (l-a-Lysophosphatidylcholine and lysophosphatidylserine) under hypoxic culture conditions for 70 days [158]. Recently, retinoic acid in combination with stem cell factor (SCF) improved IVS and differentiation of SSCs in rats by upregulating *PRTM1*, *STRA8*, c-*KIT*, *PIWIL2*, and *OCT4* gene expression [159]. Furthermore, vitamin E was effective in IVS as a reactive oxygen species (ROS) scavenger and an effective molecule to improve the yield of in vitro spermatogenesis from frozen thawed prepubertal mouse testicular tissue [160].

## 3. Other Stem Cell Types (iPSCs, ESCs, VSELs, and MSCs)

SSCs are used in this process; however, other stem cell types can also be used, particularly in patients who do not have SSCs. Therefore, other stem cells represent hope for these people to allow them to have offspring in the future. ESCs are extracted from the inner blastocyst cell mass and are considered to be pluripotent cells that can differentiate into different cell lineages. They can also provide germ cell lines in vitro. However, there is an ethical controversy regarding the use of embryonic stem cells in humans. Moreover, during differentiation, ESCs lose their plasticity [3]. ESCs differentiate into male germ-like cells in vitro, but the disadvantage is that they are not genetically related to patients [96].

On the other hand, VSELs present in humans and mice are pluripotent as they can self-renew and regenerate. Since VSELs are not affected by chemotherapy, they can contribute to germ cell development in men who have received chemotherapy as an infant [161,162].

On the other hand, iPSCs, which have the ability to be reprogrammed can be used in the replacement of ESCs, thus preventing ethical controversy. The advantages of iPSCs are an easy sampling technique, that is, somatic cells such as skin can be obtained and then reprogrammed to produce germ cells. Therefore, there is currently research underway on iPSCs to differentiate into SSCs in patients that do not have their own SSCs [3,83,132]. SSC-like cells (SSCLCs) have been generated from pluripotent stem cells and showed self-renewal over four months of culture [163].

Mesenchymal stem cells (MSCs), which are multipotent stem cells, have been used in various disciplines and diseases because of their easy isolation, culture, and immunomodulatory effects, and have a very high safety profile. MSCs have been used in male infertility, as after injection of MSCs into a busulfan-induced azoospermic rat, complete spermatogenesis was observed, and MSCs were able to restore fertility [164,165]. Interestingly, co-transplantation of SSCs and TGFβ1-treated MSCs resulted in improving the reproductive efficiency of SSC transplantation even after transplanting half the number of SSCs [166]. Because of the easy retrieval and extensive work with animals, there are some registered and recruiting clinical trials that use different kinds of stem cells (bone marrow-derived and adipose-derived MSCs) for treating non-obstructive azoospermia and impaired spermatogenesis in human subjects. However, none of these trials has posted a clinical efficiency yet. In Table 2, we summarize the up-to-date trials existing on the website clinicaltrials.gov (last accessed on 4 July 2021).

## 4. Conclusive Remarks

Treatment of male infertility caused by impaired spermatogenesis via SSC transplantation, though promising, has not attained its potential and proper implementation and still has several obstacles. There is a need for more validation of the entire process of SSC transplantation. The efficiency of cell and tissue cryopreservation must be sought with high precaution as this step is very critical and the following alternative to treat infertility depends on the efficiency and effectiveness of cryopreserved SSCs. This step is critical for preserving the future fertility of children affected with cancer before they undergo cancer treatment and for the survival of SSCs before their depletion and degeneration by chemotherapy and radiotherapy.

In addition, cryopreservation, which is a crucial and critical challenge, may be affected by the malignant contamination of the testes of children with cancer. There are major concerns about enhancing the protocol efficiency without any damage to the testicular niche [84]. The socio-cultural attitude of a community and its perspective also remains a major challenge to the wider application of the possible alternative of SSCs under question. This is mainly because adult patients who can preserve sperm obtained from ejaculated semen or TESE are not being considered for stem cell therapies. There is a lack of experience and knowledge regarding cryopreservation of testicular tissue in prepubertal patients, and few institutions have the experience, infrastructure, or regulatory approval to process and preserve testicular tissue [82].

Overall, tangible and accurate therapy options in children and adults for male infertility are still in their infancy, and artificial reproductive assisted therapy has to progress much to attain a satisfactory level of male infertility therapy, at least to date.

## Figures and Tables

**Table 1:** Milestone and trends of using spermatogonial stem cells to treat impaired spermatogenesis and non-obstructive azoospermia in different species.

Species	Level of In Vitro Culture Achieved	Time of In Vitro Culture (Year Achieved)	Culture Media Used	Reference
Human	Testicular tissue maintained	Several weeks (1970)	Eagle’ss minimum essential media (MEM)	[92]
	Propagation of SSCs but in undifferentiated	Two months propagation (2015)	StemPro-34 SFM (serum free medium)	[137]
Monkey	Maintained SSCs	Only short time (2012)	Germline culture medium	[145]
	Maintained SSCs	Effective for longer time (2017)	Stem-Pro medium	[146]
Mouse	Organ (testicular) fragment maintained	6 days (1959)	Eagle’ss MEM	[147]
	Organ (testicular) fragment maintained	4 weeks (1964)	Eagle’ss MEM	[148]
	Organ maintained and differentiation of spermatogonia to spermatocytes	2–3 weeks of culture (1964)	Eagle’ss MEM	[149]
	Differentiation of type A spermatogonia into meiotic pachytene spermatocytes	After 3 weeks (1993)	FSH supplemented Eagle’ss MEM	[150]
	Round spermatids observed (able to fertilize oocyte)	After 2 weeks of culture (2003)	Gas-liquid interface culture system	[151]
	Obtained spermatid and sperm (Produced reproductively competent offspring by microinsemination)	After 2 months (2011) [the first successful IVS]	Gas-liquid interface culture system (serum free)	[152]
	Haploid male germ cells obtained from infertile mutant mouse (Offspring produced)	Monitored growth (2012)	Agarose gel in α-MEM supplemented with KO serum replacement (KSR) or AlbuMAX	[153]
	IVS also achieved from cryopreserved testis tissue (Offspring produced)	Monitored growth (2014)	Agarose gel in α-MEM supplemented with KSR or AlbuMAX	[154]
Minipig	SSCs differentiate and develop to a post-meiotic stage	Ten days culture (2018)	MEM-α supplemented with KO serum replacement	[155]

**Table 2:** Registered clinical trials that use stem cells for treating impaired spermatogenesis.

Subjects/Cases	Intervention	Outcome	Geographic Location	Status	Trial Identifier
Azoospermic Patients	Bone Marrow Derived Mesenchymal Stem Cells	No results posted	Cairo, Egypt	Recruiting	NCT02025270
Non-obstructive Azoospermia	Bone marrow derived CD34+, CD133+, and mesenchymal stem cells	No results posted	Amman, Jordan	Recruiting	NCT02641769
Klinefelter Syndrome Azoospermia	Bone marrow Mesenchymal stem cell injection	No results posted	Cairo, Egypt	Recruiting	NCT02414295
Non-obstructive Azoospermia	Bone Marrow Derived Stem Cells	No results posted	Giza, Egypt	Recruiting	NCT02041910
Non-obstructive Azoospermia	Bone Marrow Derived Stem Cells	No results posted	Cairo, Egypt	Recruiting	NCT02008799
Azoospermia and oligozoospermia	Adipose-Derived Adult Stromal Vascular Cells	No results posted	Samara, Russian Federation	Enrolling by invitation	NCT03762967
